# The Treatment of Pelvic Inflammations through the Vagina

**Published:** 1900-03

**Authors:** 


THE TREATMENT OF PELVIC INFLAMMATIONS THROUGH THE VAGINA. By W. R. Pryor, M. D., Professor of Gynecology, New York Polyclinic, Etc. One hundred and ten illustrations. Pages, 248. Price, $2.00, net, of the publishers. Philadelphia: W. B. Saunders, 925 Walnut Street. 1899.

Professor Pryor is a leading type of the present-day surgeon, and is one of the clearest thinkers and cleanest and quickest operators in Amercia. The great number of medical gentlemen throughout the country who have attended the New York Polyclinic will appreciate this work and accord it a warm welcome. It consists of an elaboration of the author's lectures and is characterized by good work, a bold hand and a clear judgment.

The author advocates and explains a successful method of treating pelvic inflammations. Operative interference is advised in nearly every condition and the detail of the procedure is given minutely. In a word, the author tells what he thinks and does, and does not burden the reader with hereitary information. In this the work is distinctive and commendable in the highest degree.

T. J. B.

The book is highly recommended. It should be a companion to the "Surgical Complications and Sequelæ of Typhoid Fever," by Keen, issued about a year ago. T. J. B.

